# Supplementary material for: Rational design of a triple-type human papillomavirus vaccine by compromising viral-type specificity
Source: Nat Commun. 2018 Dec 18;9:5360. doi: 10.1038/s41467-018-07199-6 (PMC6299097; doi:10.1038/s41467-018-07199-6)
Supplement: Supplementary file 1 — Description of Additional Supplementary Files [file 41467_2018_7199_MOESM1_ESM.docx]

**Description of Additional Supplementary Files**

File Name: Supplementary data 1

Description: Primer sequences used in this study. The usage of each primer is also described.
