# Supplementary material for: Rational design of a triple-type human papillomavirus vaccine by compromising viral-type specificity
Source: Nat Commun. 2018 Dec 18;9:5360. doi: 10.1038/s41467-018-07199-6 (PMC6299097; doi:10.1038/s41467-018-07199-6)
Supplement: Supplementary file 2 — Supplementary Data 1 [file 41467_2018_7199_MOESM2_ESM.docx]

**Supplementary Data file | primer list**

Primers used for the clone construction of HPV33 and HPV52 pentamers

| Primer name | Primer sequence (5' to 3') |
| --- | --- |
| H33N9-C176S-F | GTAAAGGTGTTGCTAGTACTAATGCAGCACC |
| H33N9-C176S-R | GGTGCTGCATTAGTACTAGCAACACCTTTAC |
| H52N40-C204S-F | GCAAGGGCACCCCCAGCAACAACAACAGCGG |
| H52N40-C204S-R | CCGCTGTTGTTGTTGCTGGGGGTGCCCTTGC |

Primers used for the clone construction of chimeric HPV33/58 L1s

| Primer name | Primer sequence (5' to 3') |
| --- | --- |
| H33N9-58BC-F | ATTTTTCTATTAAAAGCCCTAACAACAACAAAAAAGTGTTGGTACCCAAAG |
| H33N9-58BC-R | CTTTGGGTACCAACACTTTTTTGTTGTTGTTAGGGCTTTTAATAGAAAAAT |
| H33N9-58DE-1F | ATTAGGCGTTGGCGTGAGTGGTCATCCTTACTTAAACAAATTTG |
| H33N9-58DE-1R | CAAATTTGTTTAAGTAAGGATGACCACTCACGCCAACGCCTAAT |
| H33N9-58DE-2F | GATGACACTGAAACCAGCAACAGGTATCCTGGACAAC |
| H33N9-58DE-2R | GTTGTCCAGGATACCTGTTGCTGGTTTCAGTGTCATC |
| H33N9-58DE-3F | AACAGGTATCCTGCCCAACCGGGTAGCGATAATAGGGAAT |
| H33N9-58DE-3R | ATTCCCTATTATCGCTACCCGGTTGGGCAGGATACCTGTT |
| H33N9-58EF-F | GTGTTGCTTGTAATAATAATGCAGCTGCCACTGATTGTCCACC |
| H33N9-58EF-R | GGTGGACAATCAGTGGCAGCTGCATTATTATTACAAGCAACAC |
| H33N9-58FG-F | AAGGTTCAGGAAACACCGCCGTTATTCAAAGCAGT |
| H33N9-58FG-R | ACTGCTTTGAATAACGGCGGTGTTTCCTGAACCTT |
| H33N9-58HI-F | GCACACAGGTGACCAAGGAGGGTACATACAAGAATGACAATTTTAAAG |
| H33N9-58HI-R | CTTTAAAATTGTCATTCTTGTATGTACCCTCCTTGGTCACCTGTGTGC |
| H58N35-33BC-F | CTTCAGCATCAAGAATCCCACTAACGCTAAAAAATTACTGGTGCCCAAGG |
| H58N35-33BC-R | CCTTGGGCACCAGTAATTTTTTAGCGTTAGTGGGATTCTTGATGCTGAAG |
| H58N35-33DE-1F | CCTGGGCGTCGGAATAAGCGGCCACCCCTTACTGAACAAGTTCG |
| H58N35-33DE-1R | CGAACTTGTTCAGTAAGGGGTGGCCGCTTATTCCGACGCCCAGG |
| H58N35-33DE-2F | CGACACCGAGACCGGAAACAAGTACCCCGCCCAGC |
| H58N35-33DE-2R | GCTGGGCGGGGTACTTGTTTCCGGTCTCGGTGTCG |
| H58N35-33DE-3F | AACAAGTACCCCGGACAGCCCGGCGCTGACAACAGGGAGT |
| H58N35-33DE-3R | ACTCCCTGTTGTCAGCGCCGGGCTGTCCGGGGTACTTGTT |
| H58N35-33EF-F | GGGGCAAGGGAGTAGCATGTACAAACGCTGCACCTGCCAACGACTGC |
| H58N35-33EF-R | GCAGTCGTTGGCAGGTGCAGCGTTTGTACATGCTACTCCCTTGCCCC |
| H58N35-33FG-F | CAAGGGCAGCGGCACAACAGCAAGTATCCAGAGCAGCG |
| H58N35-33FG-R | CGCTGCTCTGGATACTTGCTGTTGTGCCGCTGCCCTTG |
| H58N35-33HI-F | CCGAGGTGACCAGCGACAGCACGTACAAGAACGAGAACTTCAAGGAG |
| H58N35-33HI-R | CTCCTTGAAGTTCTCGTTCTTGTACGTGCTGTCGCTGGTCACCTCGG |

Primers used for the clone construction of chimeric HPV33/58/52 L1s

| Primer name | Primer sequence (5' to 3') |
| --- | --- |
| G-V-H33N9-58HI-52BC-F | GTCCGTTTACCAGATCCTAAT |
| G-V-H33N9-58HI-52BC-R | AGCAAGAAGTCTGGAACTACC |
| G-V-H33N9-58HI-52DE-F | TTACTTGGATGTAAGCCTCC |
| G-V-H33N9-58HI-52DE-R | CTGCCCATACTAATCGTTGTGT |
| G-V-H33N9-58HI-52EF-F | GAACTTATAAATACTATTATTG |
| G-V-H33N9-58HI-52EF-R | TGTTGGAGGCTTACATCC |
| G-V-H33N9-58HI-52FG-F | GTATTTGTTACTGTGGTAGAT |
| G-V-H33N9-58HI-52FG-R | AGTCATTTTTAAATAATCTGGAT |
| G-H33N9-58HI-52BC-F | GCTGGTAGTTCCAGACTTCTTGCTGTGGGCCACCCCTACTTCAGC |
| G-H33N9-58HI-52BC-R | TATTAGGATCTGGTAAACGGACCCTGAACACCCTGTACTGCAG |
| G-H33N9-58HI-52DE-F | ACGATTAGTATGGGCATGTGTAGGCCTGGAGATCGGCAGGGGC |
| G-H33N9-58HI-52DE-R | TTGGAGGCTTACATCCAAGTAAGCACAGCTGGGTCTGCTTGTAG |
| G-H33N9-58HI-52EF-F | TACTTGGATGTAAGCCTCCAACAGGCGAGCACTGGGGCAAGGGCAC |
| G-H33N9-58HI-52EF-R | TCAATAATAGTATTTATAAGTTCCAGGGGGGGGCAGTCGCCGGGGT |
| G-H33N9-58HI-52FG-F | TCCAGATTATTTAAAAATGACTAGCGAGCCCTACGGCGACAGCCT |
| G-H33N9-58HI-52FG-R | TATCTACCACAGTAACAAATACCTGGTTGCCCCAGCAGATGCCG |
| G-V-H58N35-33BC-52DE-F | CTGATCGGCTGCAAGCCCCCCAC |
| G-V-H58N35-33BC-52DE-R | CACGCAGGCCCACACCAGCC |
| G-V-H58N35-33BC-52EF-F | GAGCTGTTCAACAGCATCATCGAGG |
| G-V-H58N35-33BC-52EF-R | GGTGGGGGGCTTGCAGCCGATC |
| G-V-H58N35-33BC-52FG-F | ATCGTGACCAGCGAGAGCCAGC |
| G-V-H58N35-33BC-52FG-R | CTTCAGGTAGTCGGGGTACTTGC |
| G-V-H58N35-33BC-52HI-F | GTGAGGCACGTGGAGGAGTACG |
| G-V-H58N35-33BC-52HI-R | GGTGCACAGGGTCATGTTGGTG |
| G-H58N35-33BC-52DE-F | GAGGCTGGTGTGGGCCTGCGTGGGCCTGGAGATCGGCAGG |
| G-H58N35-33BC-52DE-R | TGGGGGGCTTGCAGCCGATCAGGCACAGCTGGGTCTGCTTG |
| G-H58N35-33BC-52EF-F | TGATCGGCTGCAAGCCCCCCACCGGCGAGCACTGGGGCAAGGG |
| G-H58N35-33BC-52EF-R | TCGATGATGCTGTTGAACAGCTCCAGGGGGGGGCAGTCGCCG |
| G-H58N35-33BC-52FG-F | GCAAGTACCCCGACTACCTGAAGATGGCCAGCGAGCCCT |
| G-H58N35-33BC-52FG-R | GCTGGCTCTCGCTGGTCACGATGCTGCCGCTGGGGGTG |
| G-H58N35-33BC-52HI-F | CACCAACATGACCCTGTGCACCGAGGTGAAGAAGGAGAG |
| G-H58N35-33BC-52HI-R | CGTACTCCTCCACGTGCCTCACGTACTCCTTGAAGTTCTCG |

Primers used for the clone construction of chimeric HPV18/45/59 L1s

| Primer name | Primer sequence (5' to 3') |
| --- | --- |
| H45N27-18BC-F | CCTACTTCAGGGTGCCAGCCGGCGGCGGCAACAAGCAGGACATACCCAAGGTG |
| H45N27-18BC-R | CACCTTGGGTATGTCCTGCTTGTTGCCGCCGCCGGCTGGCACCCTGAAGTAGG |
| G-V-H45N27-18BC-59HI-F | CAGTACAGCAGGCACGTGGAGGAGT |
| G-V-H45N27-18BC-59HI-R | CAGGGTCAGGTTGGTGCTCC |
| G-H45N27-18BC-59HI-F | AGGAGCACCAACCTGACCCTGTGCGCCAGCACCACCAGCAGCAT |
| G-H45N27-18BC-59HI-R | ACTCCTCCACGTGCCTGCTGTACTGCTTGAAGCTGGTGGGGGTG |

Primers used for the clone construction of chimeric HPV56/66/53 L1s

| Primer name | Primer sequence (5' to 3') |
| --- | --- |
| G-V-H66N5-56HI-F | GGCGGCGTTGATGGTCATGTT |
| G-V-H66N5-56HI-R | ATCAACCAGTACCTGAGGCAC |
| G-H66N5-56HI-F | AACATGACCATCAACGCCGCCACCGAGCAGCTGTCCAAGTAC |
| G-H66N5-56HI-R | GTGCCTCAGGTACTGGTTGATCTTCCGGGCGTCGTACTTGGA |
| G-V-H66N5-56HI-53FG-F | GTGCCTGGCGAACAGCTGCTC |
| G-V-H66N5-56HI-53FG-R | GCCACCCCCAGCGGCAGCATG |
| G-H66N5-56HI-53FG-F | GAGCAGCTGTTCGCCAGGCACTTCTTCAACAGGGCCGGCGTG |
| G-H66N5-56HI-53FG-R | CATGCTGCCGCTGGGGGTGGCCACGTACACGCTGCTGGGGGG |

Primers used for the clone construction of HPV39/68/70 L1s

| Primer name | Primer sequence (5' to 3') |
| --- | --- |
| G-V-H39N15-68FG-F | GTGGCGGGCGAAGAGCTGCTCGCG |
| G-V-H39N15-68FG-R | TGTCCAAGCCCATCCGGCAGCATG |
| G-H39N15-68FG-F | CGCGAGCAGCTCTTCGCCCGCCACTTCTGGAACAGAGGCGGCATG |
| G-H39N15-68FG-R | CATGCTGCCGGATGGGCTTGGACAGTACACGTAGCTGCTGGGTGT |
| G-V-H39N15-68FG-70HI-F | GCTTGTGCTCAGGGTGAAATT |
| G-V-H39N15-68FG-70HI-R | TTCAAGGAGTACACACGCCAC |
| G-H39N15-68FG-70HI-F | AATTTCACCCTGAGCACAAGCACCGAAACAGCCATACCTGCT |
| G-H39N15-68FG-70HI-R | GTGGCGTGTGTACTCCTTGAACTTTGTAGGGCTATATACAGC |

Primers used for the clone construction of chimeric HPV33/59 and H58/59 L1s

| Primer name | Primer sequence (5' to 3') |
| --- | --- |
| H33N9-59HI-F | AGCATCCCCAACGTGTACACCCCCACCAGCTTTAAAGAATATATAAG |
| H33N9-59HI-R | GTACACGTTGGGGATGCTGCTGGTGGTCTGTGTGCATAAAGTC |
| H58N35-59BC-F | GCCGTTGCCGCCCTTGGGCACCTTGAACTGAAGTAGGGGTTGCCCAC |
| H58N35-59BC-R | CCCAAGGGCGGCAACGGCAGGCAGGACGTGCCCAAGGTGAGCGGCC |

Primers used for the clone construction of HPV33/58 single-point L1 mutatants

| Primer name | Primer sequence (5' to 3') |
| --- | --- |
| H33N9-EF50K-F | ATGCACACAGGTAACTAAAGACAGTACATAT |
| H33N9-EF50K-R | ATATGTACTGTCTTTAGTTACCTGTGTGCAT |
| H33N9-D351E-F | CACAGGTAACTAGTGAAAGTACATATAAAAATG |
| H33N9-D351E-R | CATTTTTATATGTACTTTCACTAGTTACCTGTG |
| H33N9-EF52G-F | CAGGTAACTAGTGACGGTACATATAAAAATG |
| H33N9-EF52G-R | CATTTTTATATGTACCGTCACTAGTTACCTG |
| H33N9-E357D-F | GTACATATAAAAATGATAATTTTAAAGAATAT |
| H33N9-E357D-R | ATATTCTTTAAAATTATCATTTTTATATGTAC |
| H58N35-S80N-F | CTTCAGCATCAAGAACCCCAACAACAACAAG |
| H58N35-S80N-R | CTTGTTGTTGTTGGGGTTCTTGATGCTGAAG |
| H58N35-N82T-F | GCATCAAGAGCCCCACCAACAACAAGAAGGT |
| H58N35-N82T-R | ACCTTCTTGTTGTTGGTGGGGCTCTTGATGC |
| H58N35-N84A-F | AGAGCCCCAACAACGCCAAGAAGGTGCTGGT |
| H58N35-N84A-R | ACCAGCACCTTCTTGGCGTTGTTGGGGCTCT |
| H58N35-V87L-F | CAACAACAAGAAGCTGCTGGTGCCCAAGGTG |
| H58N35-V87L-R | CACCTTGGGCACCAGCAGCTTCTTGTTGTTG |
