# Supplementary material for: Rational design of a triple-type human papillomavirus vaccine by compromising viral-type specificity
Source: Nat Commun. 2018 Dec 18;9:5360. doi: 10.1038/s41467-018-07199-6 (PMC6299097; doi:10.1038/s41467-018-07199-6)
Supplement: Supplementary file 3 — Supplementary Information [file 41467_2018_7199_MOESM3_ESM.pdf]

Supplementary information

Rational Design of a Triple-Type Human Papillomavirus Vaccine by Compromising Viral-Type Specificity

Li et al.

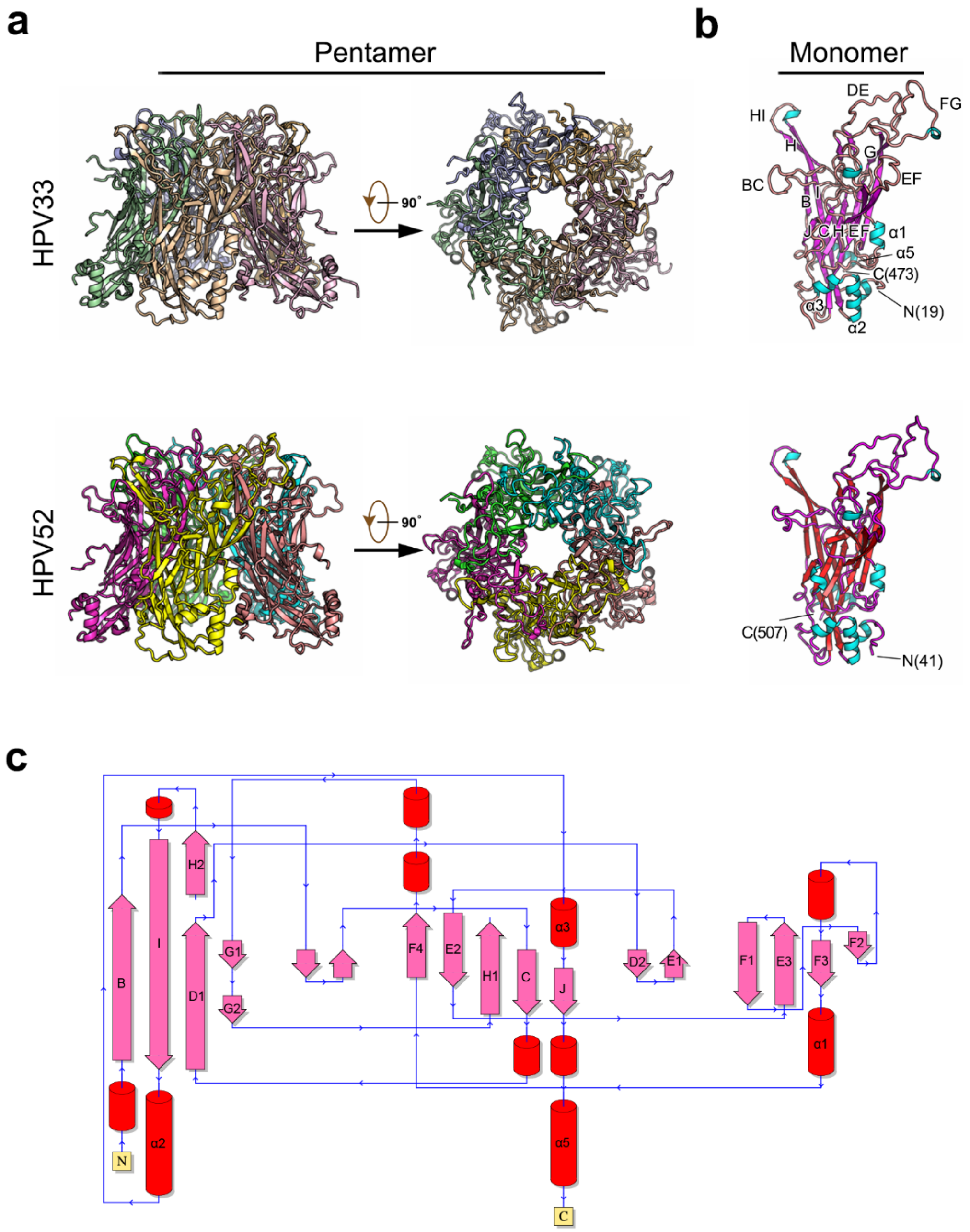

Supplementary Figure 1. Crystal structures of HPV33 and HPV52 pentamers.

a Side-view (left) and top-view (right) of crystal structures of HPV33 (upper) and HPV52 (lower) pentamer with five monomers in different colors. **b** Crystal structures of HPV33 (upper) and HPV52 (lower) monomers colored by different secondary structural elements (see labels). **c** Topology diagram of the major capsid protein (L1) of HPV. β-strands, α-helices, and connecting loops are represented by pink arrows, red cylinders and blue lines, respectively.

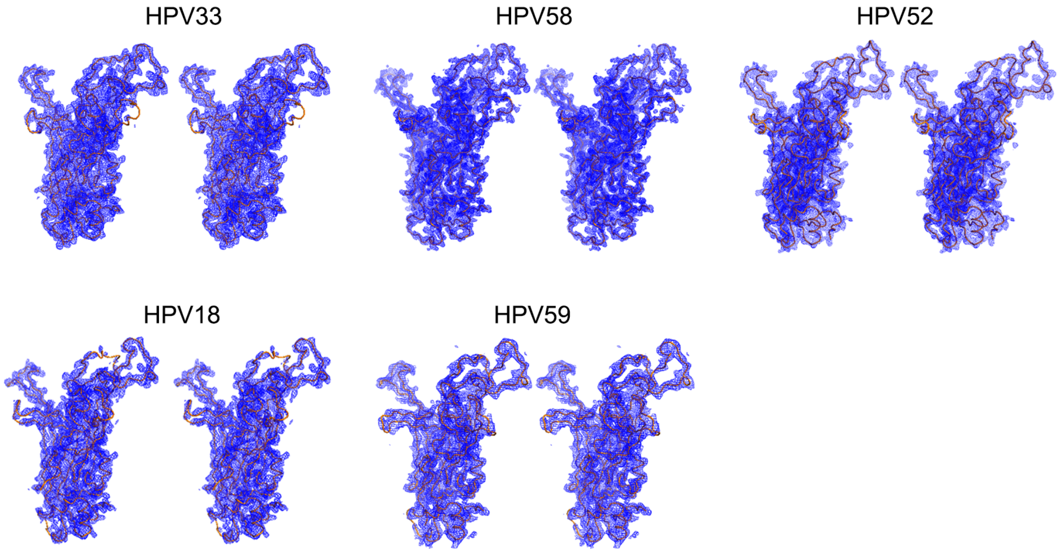

Supplementary Figure 2. L1 structures of different HPV types. Stereo images of main chain models within the electron density maps of the crystal structures of L1 proteins of HPV33, HPV58 (PDB code: 5Y9E[1]), HPV52, HPV18 (PDB code: 2R5I[2]), and HPV59 (PDB code: 5J6R[3]). The main chains of L1 monomers are shown as orange ribbons within the 2Fo-Fc electron density maps for the refined structure (blue), contoured at 1σ.

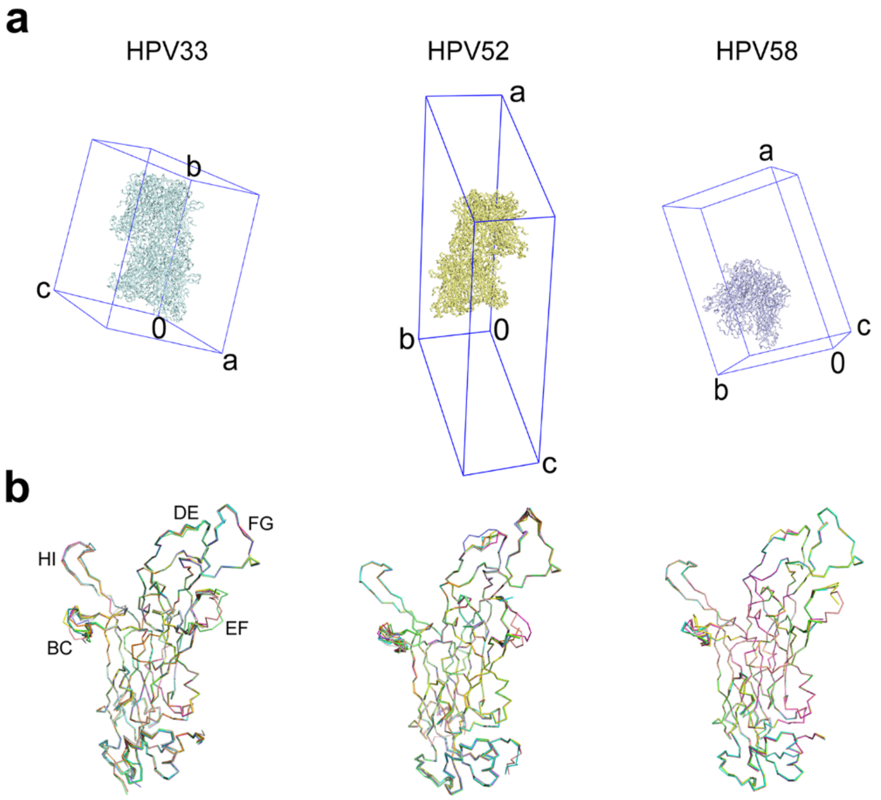

Supplementary Figure 3. Superimposition of monomers in one asymmetric unit (ASU) of the crystal structures of HPV33, HPV52, and HPV58.

a Crystal packing of pentamers of HPV33, -52 and -58. **b** Superimposition of monomers shows slight structural variances in the five surface loops among different monomers within one ASU of the crystal structure.

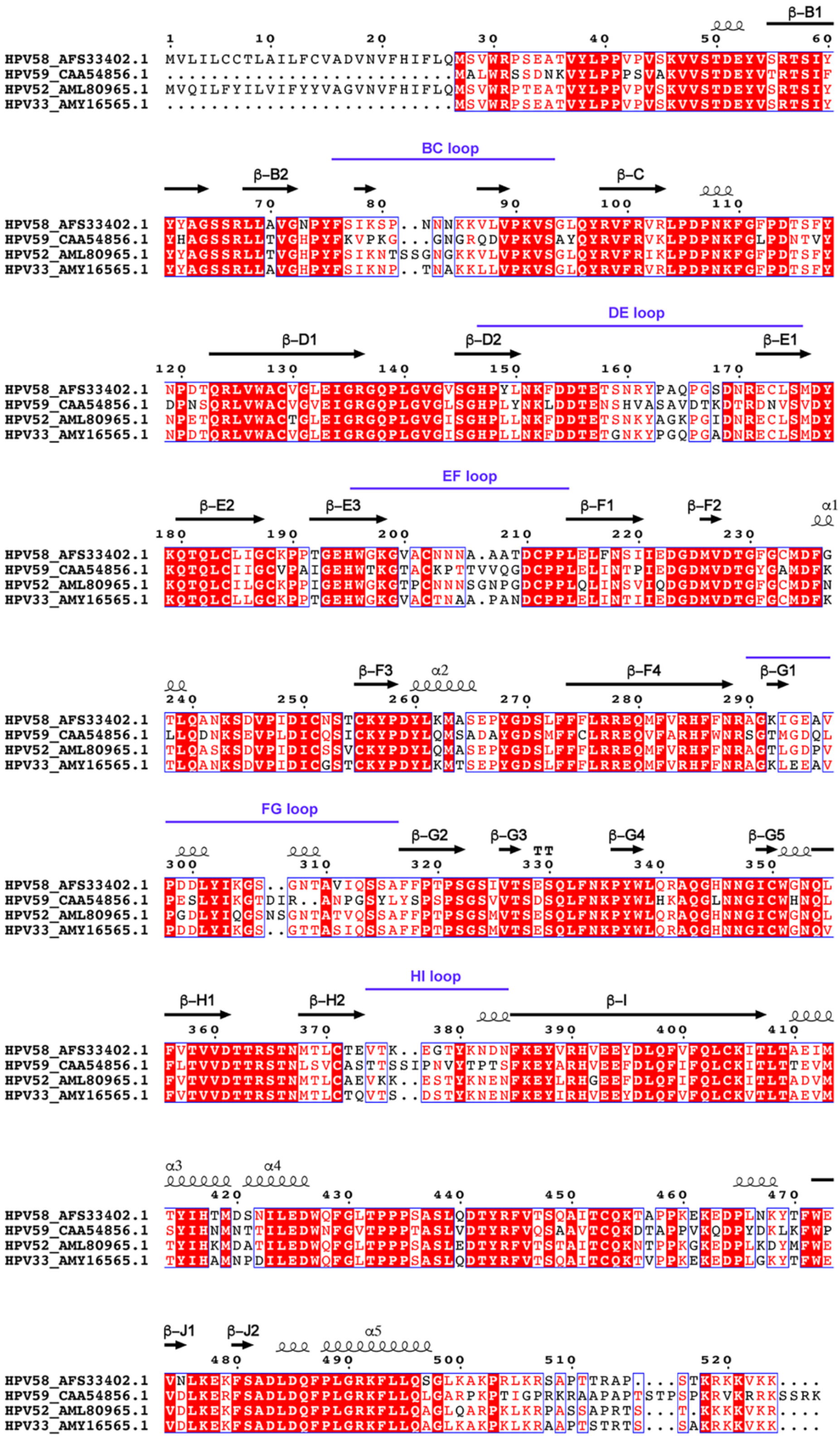

Supplementary Figure 4. Sequence alignment of L1 proteins of HPV58, -59, -52, and -33. Secondary structural elements are shown above the sequences. The six variable regions (BC, DE, EF, FG, HI, and C-terminal arm) are also labeled.

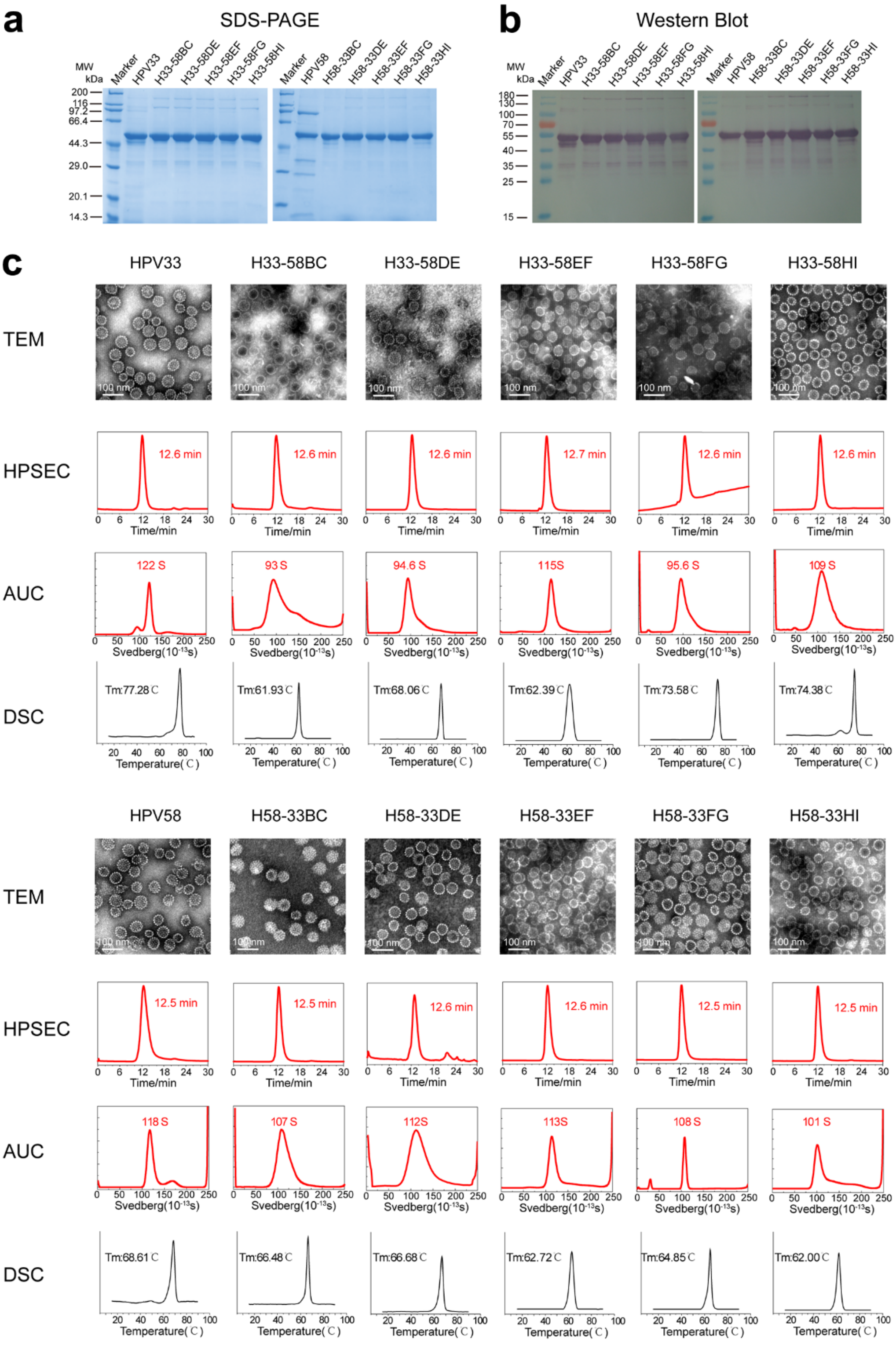

Supplementary Figure 5. Characterization of chimeric HPV33/58 L1 proteins and VLPs. The mutants and WT L1 proteins were subjected to reducing SDS-PAGE **a** and western blotting **b** with a wide-spectrum linear mAb 4B3. **c** Transmission electron microscopy (TEM) images, high-performance size-exclusion chromatography (HPSEC) profiles, analytical ultracentrifugation sedimentation (AUC) profiles, and differential scanning calorimetry (DSC) profiles of the mutants and WT L1 VLPs.

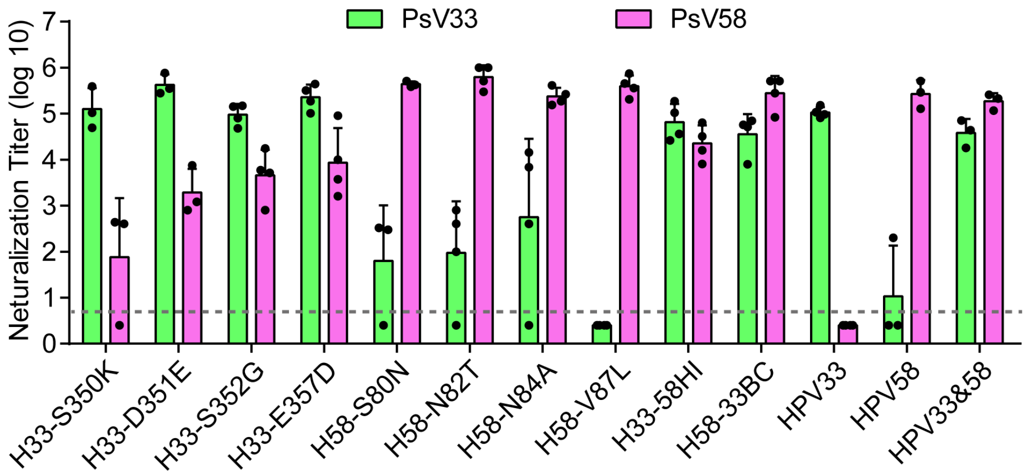

Supplementary Figure 6. Immunogenicity of single-point substituted HPV33/58 chimeric VLPs.

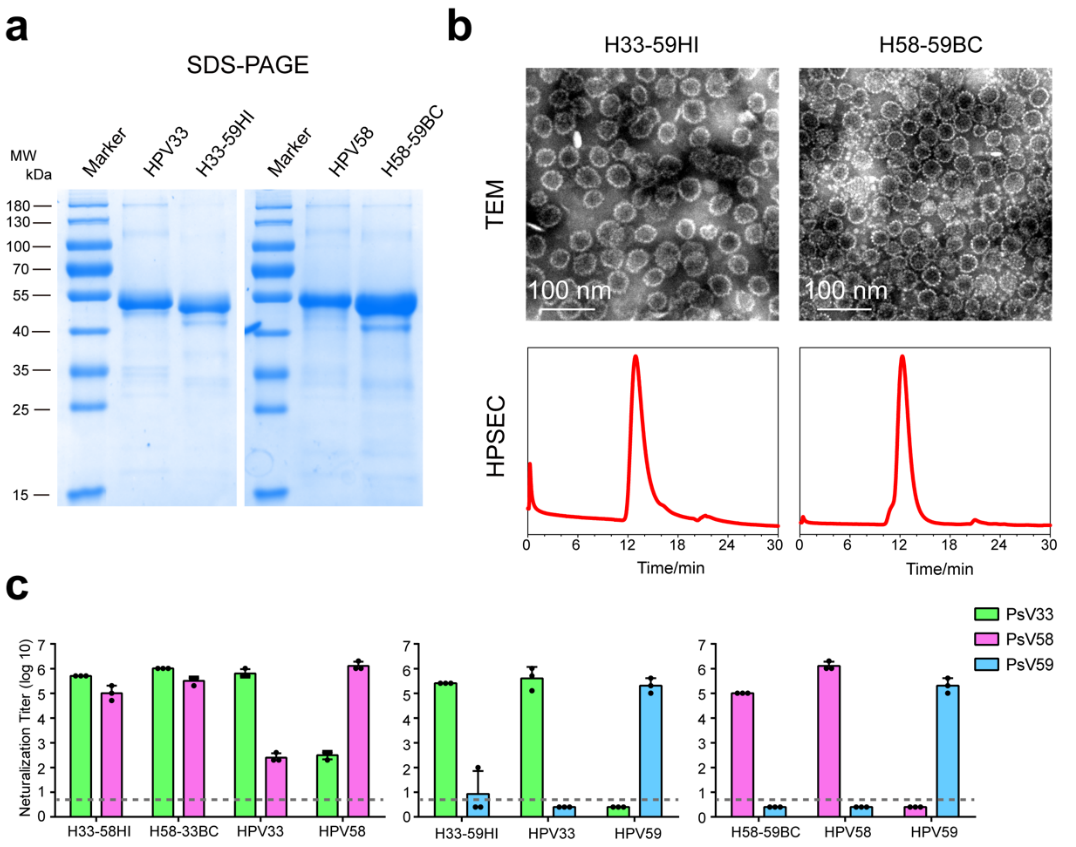

Supplementary Figure 7. Immunogenicity of chimeric VLPs H33-59HI and H58-59BC. The purity of mutated L1 proteins and the particle morphology were characterized by reducing SDS-PAGE **a** and transmission electron microscopy (TEM) images, or high-performance size-exclusion chromatography (HPSEC) profiles **b**, respectively. **c** Neutralization titers of immune sera (at week 8) in BALB/c mice immunized with 100 μg/dose of chimeric HPV33/58 (left), HPV33/59 (middle) and HPV58/59 VLPs (right) formulated with Freund's adjuvant at weeks 0, 2 and 4.

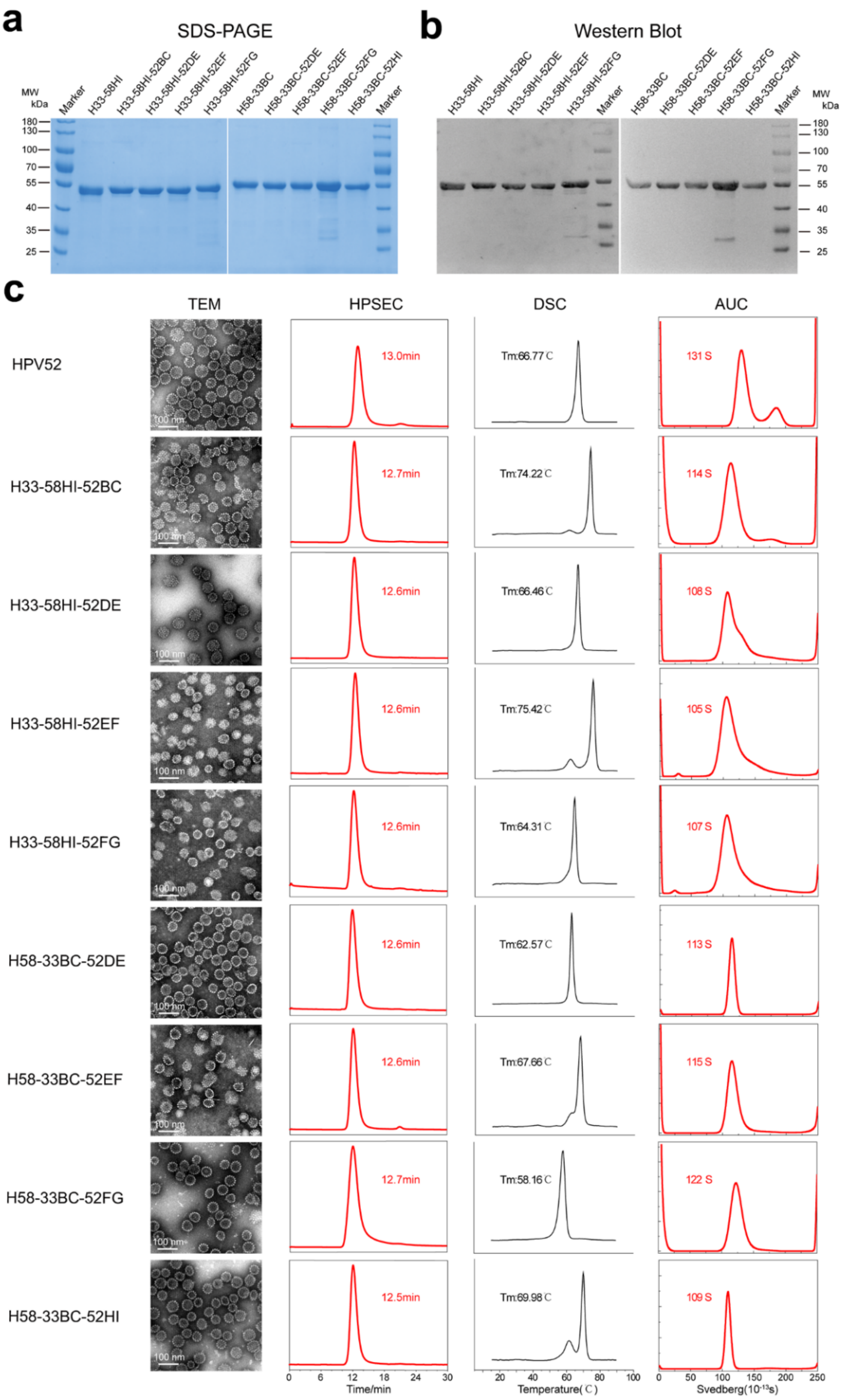

Supplementary Figure 8. Characterization of triple-type chimeric HPV33/52/58 L1 proteins and VLPs. The mutants and WT L1 proteins were subjected to reducing SDS-PAGE **a** and western blotting **b** with a wide-spectrum linear mAb 4B3. **c** TEM, HPSEC, AUC and DSC of the mutants and WT L1 VLPs.

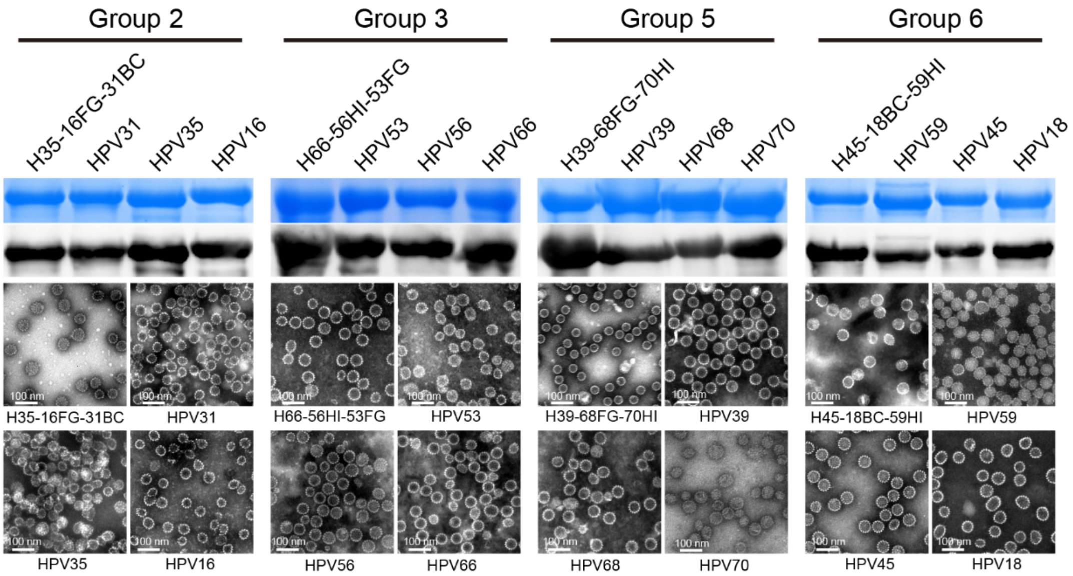

Supplementary Figure 9. Characterization of another four triple-type chimeric L1 proteins. The mutants and WT L1 proteins of Groups 2, -3, -5, and -6 were subjected to reducing SDS-PAGE, western blotting with a wide-spectrum linear mAb 4B3, and transmission electron microscopy images.

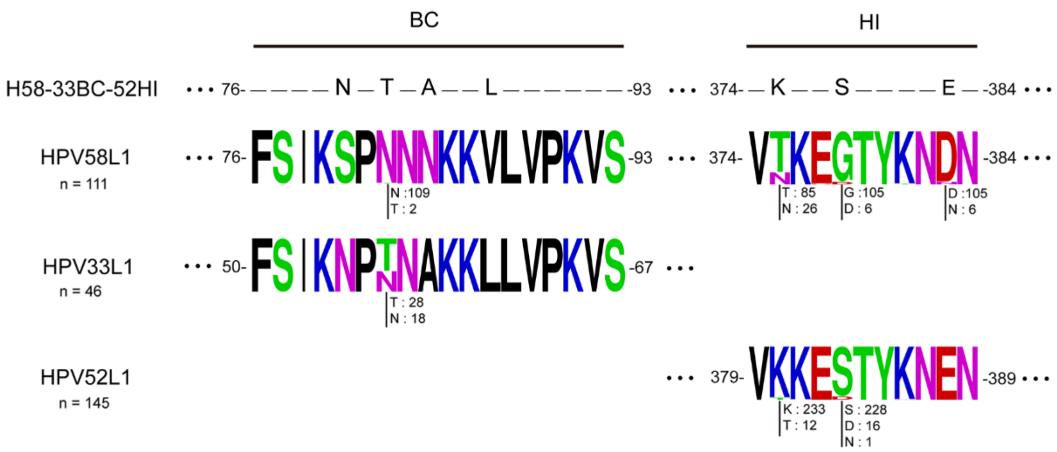

Supplementary Figure 10. Amino acid sequence conservation analyses on BC and HI loops of HPV33, HPV52, and HPV58. Sequences of HPV58 (*n* = 111), HPV33 (*n* = 46), and HPV52 (*n* = 145) subtypes were, respectively, aligned using MEGA5 and the aligned sequences then run in the WebLogo tool (http://weblogo.berkeley.edu/logo.cgi). Each column of the alignment is represented by a stack of letters, with the height of each letter proportional to the observed frequency of the corresponding amino acid, and the overall height of each stack corresponds to the sequence conservation at that position. The numbers below the exchanged residues in the BC loop (between HPV33 and HPV58) or the HI loop (between HPV52 and HPV58) indicate the frequency of the corresponding amino acids. The sequences for HPV33, HPV52 and HPV58 were downloaded from the NCBI database.

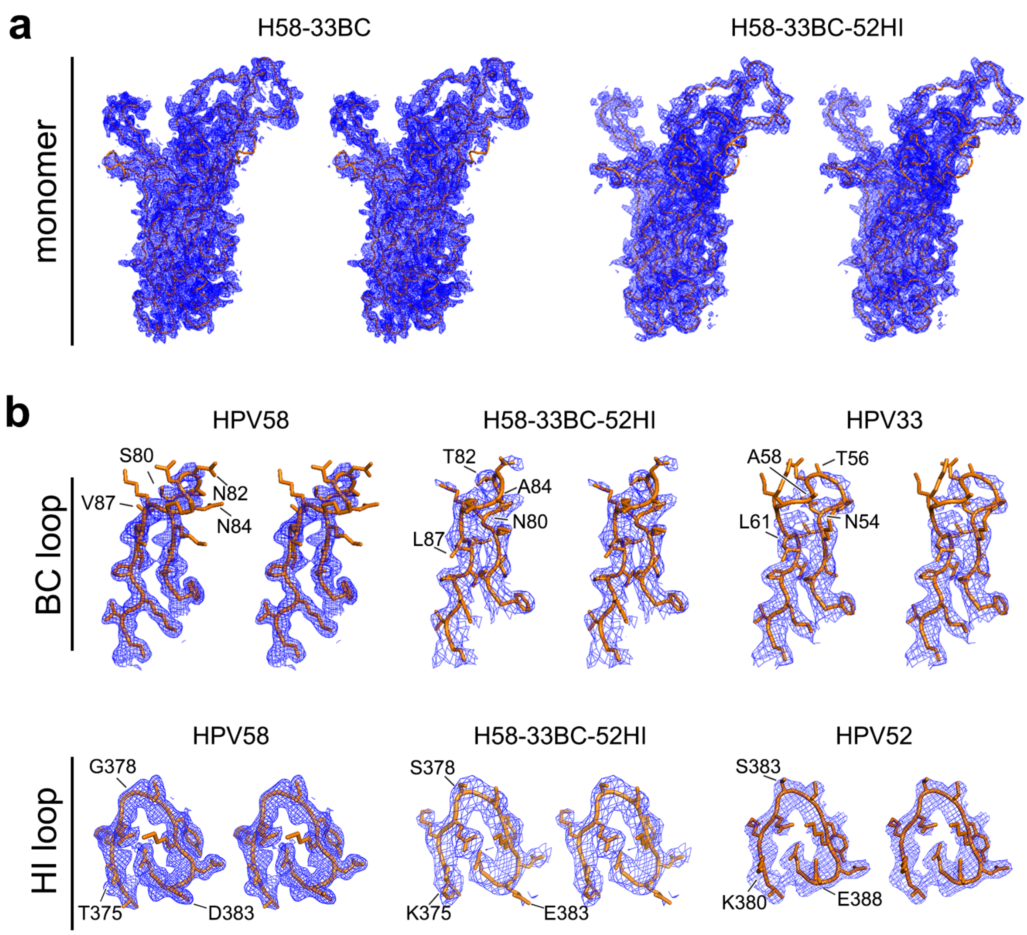

Supplementary Figure 11. Crystal structures of chimeric L1 pentamers H58-33BC and H58-33BC-52HI. a Stereo images of main chain models within electron density maps of the crystal structures of mutant L1 proteins of H58-33BC and H58-33BC-52HI. The main chains of the L1 monomers are shown as orange ribbons within the 2Fo-Fc electron density maps for the refined structures (blue), contoured at 1σ. **b** Structural comparison of the BC loops and HI loops of WT and mutants. Stereo images of sidechains of the residues in the HI loop are shown in stick form within the 2Fo-Fc electron density map for the refined structure (blue), contoured at 1σ.

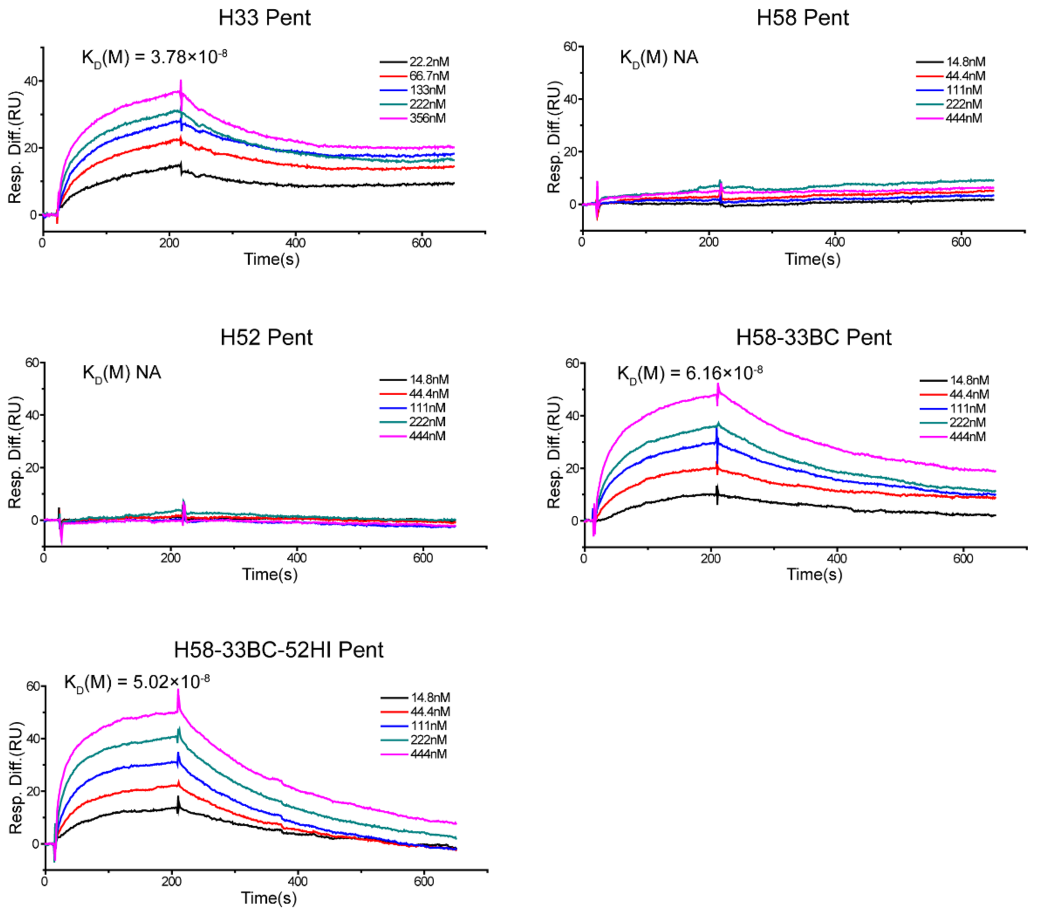

Supplementary Figure 12. WT or chimeric pentamers and mAb 4E5 binding curves in surface plasma resonance (SPR). Five concentrations of pentamer (serial dilution) were injected onto a chip bound with mAb 4E5. These binding curves were used to calculate affinity constants. The experiments were repeated twice and the results were consistent.

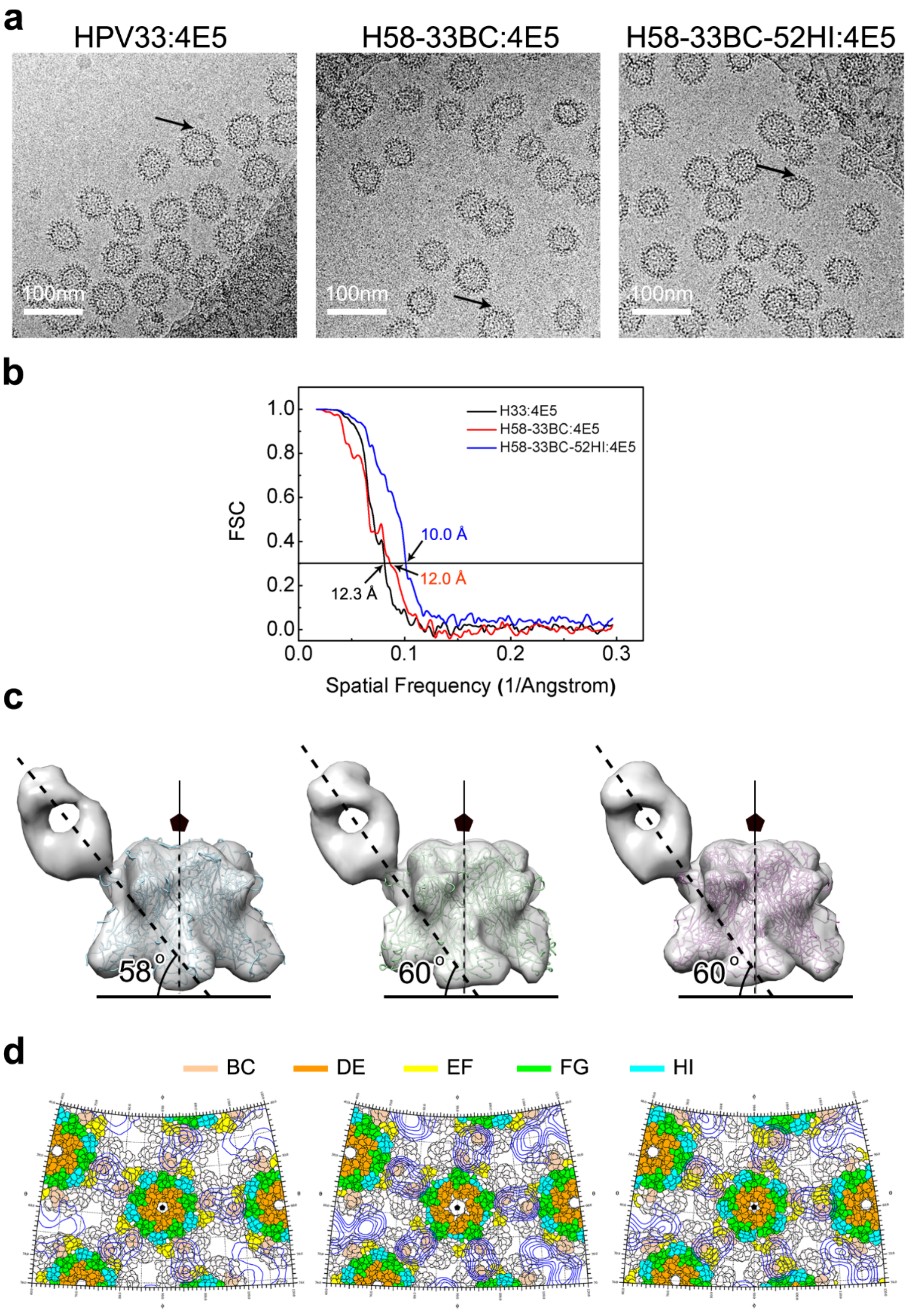

Supplementary Figure 13. Cryo-EM structures of VLPs-Fab of HPV33:4E5, H58-33BC:4E5, and H58-33BC-52HI:4E5.

(a) Representative micrographs of HPV33:4E5 (left), H58-33BC:4E5 (middle) and H58-33BC-52HI (right). The black arrows indicate the VLPs-bound Fabs. Scale bar = 100 nm.

(b) Resolution evaluation of the cryo-EM reconstructions by Fourier shell correlation (FSC) at 0.3 criterion to ensure the reliability of the estimated resolution, as only ~700-900 particles (Supplementary Table 7) were included in the final reconstructions. **(c)** Binding configurations of HPV33:4E5 (left), H58-33BC:4E5 (middle), and H58-33BC-52HI (right). mAb 4E5 obliquely bind to the pentamer surface of HPV33:4E5, H58-33BC:4E5, and H58-33BC-52HI:4E5, with tilt angles of 58°, 60°, and 60°, respectively. 5-fold axes of icosahedral capsid are indicated as perpendicular lines marked with solid pentagon insignia. For the purposed of comparability, the cryoEM maps at higher resolution were low-pass filtered to 12.3 Å. **(d)** Global view of the roadmaps of HPV33:4E5 (left), HPV58-33BC:4E5 (middle) and HPV58-33BC-52HI (right) surface, projected by an area surrounding a 5-fold vertex defined with a polar angle θ range of 40° to 76° and a polar angle ϕ range of 60° to 120°. Color scheme for surface residues are according to surface loops (BC, amber; DE, orange; EF, yellow; FG, green and HI, Cyan). Blue contour lines were drawn according to the projection of the Fab-4E5 entity with a density greater than 1 and within a radial section of the VLP-4E5 complex ranging from 310 Å to 360 Å[4]. The icosahedral symmetry axes are labeled.

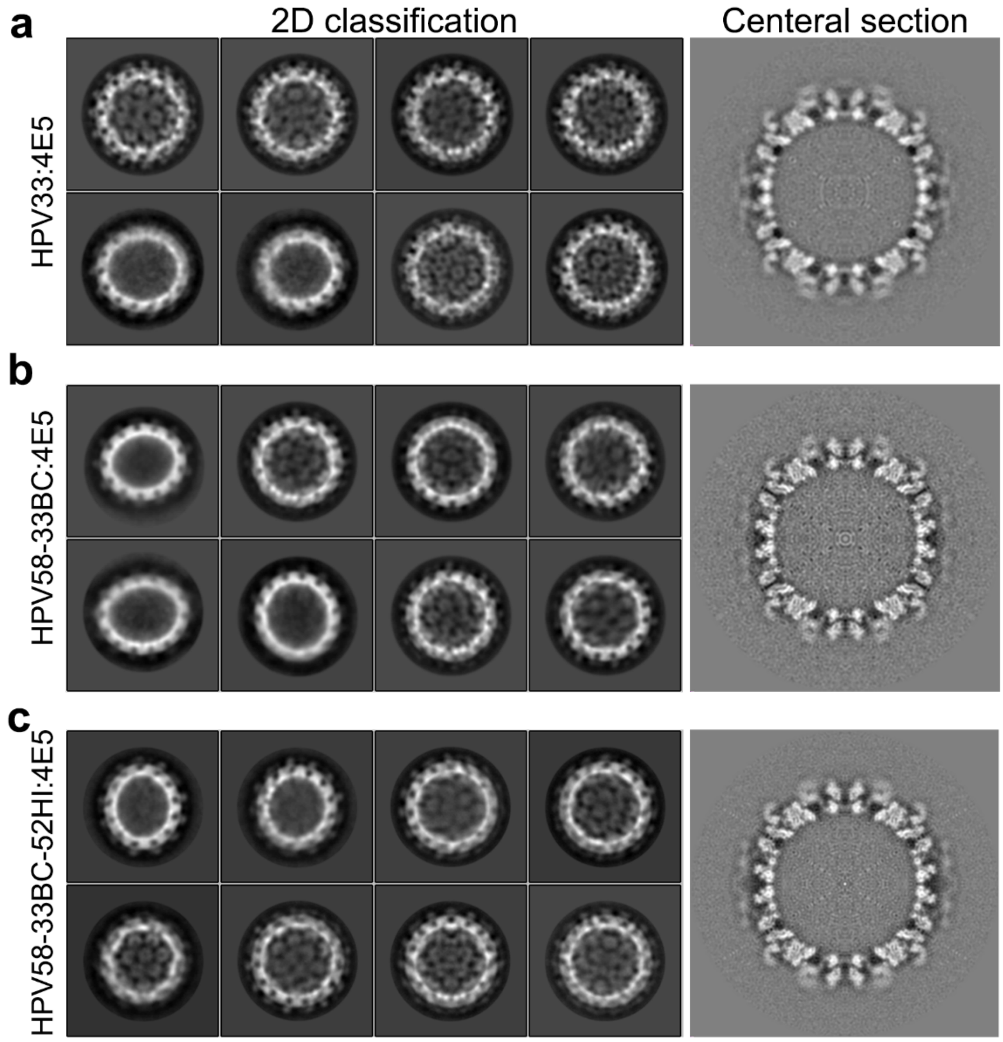

Supplementary Figure 14. Image processing of HPV33:4E5, H58-33BC:4E5, and H58-33BC-52HI:4E5. Representative 2D class averages of particles (left panel) and central sections of density maps (right panel) of three different VLP:4E5 complexes, **a** HPV33:4E5, **b** HPV58-33BC:4E5, **c** HPV58-33BC-52HI:4E5.

Supplementary Table 1. Estimates of evolutionary divergence between L1 sequences of 20 HPV types.

	6	11	16	18	26	31	33	35	39	45	51	52	53	56	58	59	66	68	69	70
6																				
11	0.08																			
16	0.46	0.46																		
18	0.59	0.63	0.55																	
26	0.57	0.60	0.51	0.50																
31	0.45	0.43	0.21	0.59	0.50															
33	0.48	0.50	0.27	0.61	0.55	0.28														
35	0.46	0.44	0.20	0.59	0.56	0.21	0.28													
39	0.58	0.61	0.58	0.29	0.48	0.61	0.63	0.60												
45	0.57	0.60	0.56	0.13	0.50	0.60	0.61	0.59	0.32											
51	0.65	0.65	0.53	0.55	0.28	0.57	0.62	0.58	0.52	0.56										
52	0.47	0.45	0.26	0.59	0.53	0.26	0.17	0.27	0.62	0.59	0.58									
53	0.58	0.57	0.55	0.51	0.47	0.55	0.54	0.59	0.53	0.51	0.47	0.58								
56	0.61	0.60	0.59	0.51	0.46	0.59	0.57	0.61	0.54	0.53	0.53	0.63	0.23							
58	0.46	0.50	0.28	0.60	0.56	0.30	0.10	0.31	0.63	0.60	0.62	0.18	0.52	0.57						
59	0.63	0.63	0.57	0.27	0.53	0.59	0.62	0.56	0.31	0.26	0.54	0.61	0.53	0.54	0.63					
66	0.61	0.58	0.57	0.53	0.49	0.58	0.58	0.62	0.56	0.57	0.55	0.63	0.24	0.12	0.60	0.56				
68	0.59	0.61	0.60	0.31	0.49	0.62	0.66	0.62	0.13	0.30	0.51	0.65	0.52	0.53	0.67	0.30	0.56			
69	0.59	0.60	0.50	0.49	0.09	0.49	0.59	0.55	0.48	0.49	0.29	0.54	0.47	0.49	0.59	0.52	0.51	0.51		
70	0.58	0.59	0.61	0.28	0.46	0.62	0.64	0.62	0.16	0.29	0.49	0.63	0.51	0.53	0.65	0.29	0.56	0.14	0.46	

Supplementary Table 2. The pairwise root mean square deviations (RMSDs) of different regions between L1 models of five HPV types

1st Type			2nd Type				
			33	58	52	59	18
33	overall		–	0.23	0.43	0.87	0.83
	core region		–	0.22	0.31	0.53	0.46
	variable region		–	0.53	0.77	1.81	1.84
		BC	–	0.96	0.98	2.54	3.50
		DE	–	0.47	0.63	2.24	1.67
		EF	–	0.71	1.63	2.75	1.98
		FG	–	0.31	0.52	1.10	1.12
		HI	–	0.36	0.49	1.20	1.20
58	overall		–	–	0.43	0.93	0.89
	core region		–	–	0.33	0.53	0.47
	variable region		–	–	0.74	2.02	2.06
		BC	–	–	1.01	3.00	4.12
		DE	–	–	0.53	2.27	1.64
		EF	–	–	1.65	3.53	2.61
		FG	–	–	0.52	1.20	1.22
		HI	–	–	0.44	1.21	1.19
52	overall		–	–	–	0.83	0.79
	core region		–	–	–	0.50	0.42
	variable region		–	–	–	1.76	1.77
		BC	–	–	–	2.75	3.89
		DE	–	–	–	2.18	1.57
		EF	–	–	–	1.43	1.21
		FG	–	–	–	1.16	1.21
		HI	–	–	–	1.17	1.12
59	overall		–	–	–	–	0.64
	core region		–	–	–	–	0.42
	variable region		–	–	–	–	1.31
		BC	–	–	–	–	2.93
		DE	–	–	–	–	1.12
		EF	–	–	–	–	1.64
		FG	–	–	–	–	0.59
		HI	–	–	–	–	0.64

Supplementary Table 3. Characterization of HPV33 and HPV58 mAb panel and antigenicity assay of HPV33/58 chimeras.

Immunogen for antibody generation (VLP)	mAbs	Isotype	ELISA /Neutralization titer (Log_{10})		mAb reactivity of WT HPV33, HPV58 and HPV33/58 chimeras (EC_{50}, ng)											
			HPV33	HPV58	HPV33	HPV58	H33-58BC	H33-58DE	H33-58EF	H33-58FG	H33-58HI	H58-33BC	H58-33DE	H58-33EF	H58-33FG	H58-33HI
HPV33	4E5	IgG1	6.6/5.5	1.7/ -	23.4	>10000	1443.0	2.3	16.7	9.0	3.3	16.7	585.1	>10000	>10000	>10000
	7C12	IgG1	5.5/1.0	2.1/ -	123.1	>10000	51.6	54.9	38.6	79.9	50.9	7113.0	5396.0	>10000	>10000	6808.0
	11A3	IgG1	5.0/1.0	1.0/ -	638.6	>10000	65.9	33.8	36.2	41.2	38.9	>10000	>10000	7546.0	>10000	>10000
	11F4	IgG1	5.0/1.0	1.7/ -	173.9	>10000	31.8	22.4	34.4	25.6	56.1	>10000	>10000	>10000	>10000	>10000
	C2C3	IgG2a	7.4/6.0	1.7/ -	8.4	>10000	>10000	3.4	18.5	3.2	2.8	18.8	1434.0	306.9	>10000	>10000
	C14A2	IgG2b	5.5/3.9	2.2/ -	66.8	>10000	>10000	17.4	320.8	62.5	39.0	134.8	4889.0	7306.0	>10000	>10000
	C16D7	IgG1	5.0/4.2	1.7/ -	483.8	>10000	>10000	5819.0	267.3	302.8	460.5	855.7	6.5	7110.0	>10000	>10000
	9A3	IgG1	6.0/2.0	2.7/ -	77.7	>10000	>10000	>10000	>10000	>10000	9496.0	8047.0	6787.0	9709.0	>10000	>10000
	7A3	IgG3	5.0/4.1	1.0/ -	2394.0	>10000	>10000	>10000	1230.0	1964.0	>10000	>10000	>10000	>10000	>10000	>10000
HPV58	4A2	IgG1	2.2/ -	5.0/3.8	>10000	100.4	>10000	421.8	63.0	18.3	7532.0	24.5	13.3	214.4	70.5	483.7
	13A4	IgG2b	2.2/ -	5.5/3.5	>10000	57.4	>10000	94.6	19.1	10.7	7560.0	29.4	30.3	>10000	2.0	23.2
	5H2	IgG1	2.2/ -	6.0/3.8	>10000	20.0	>10000	596.1	16.5	6.8	944.8	6.3	13.9	333.6	9.0	19.2
	10B11	IgG2b	1.7/ -	5.5/4.4	>10000	271.8	>10000	1598.0	275.4	228.3	>10000	19.2	67.4	34.9	349.1	225.2
	5G9	IgG2a	2.2/ -	5.5/5.0	>10000	39.0	>10000	5.4	>10000	>10000	>10000	10.5	7537.0	11.6	15.8	14.9
	A15A9	IgG2b	2.2/ -	4.5/3.5	>10000	264.4	>10000	90.7	>10000	>10000	>10000	29.9	2819.0	34.4	159.0	33.2
	A10B8	IgG2b	1.0/ -	5.0/4.4	>10000	259.3	>10000	793.2	>10000	>10000	>10000	21.2	1077.0	11.5	45.7	9.4
	1D4	IgG2b	1.7/ -	5.0/5.0	>10000	107.0	>10000	28.5	>10000	>10000	>10000	43.8	4592.0	18.9	57.1	48.7
	3E4	IgG2b	1.0/ -	5.6/4.4	>10000	182.8	>10000	65.0	>10000	>10000	>10000	53.7	4231.0	28.0	192.0	72.0

Supplementary Table 4. Half-Effective Dose (ED_{50}) in Mice for Aluminum Adjuvant-Containing Chimeric or WT VLPs of HPV33 and HPV58.

Antigen (VLP)	Dose (μg)	PsV33		PsV58	
		Seroconversion no./ inoculated no.	ED_{50} (μg)*	Seroconversion no./ inoculated no.	ED_{50} (μg)
HPV33	0.300	8/8	0.066	0/8	>0.3
	0.100	5/8		0/8	
	0.033	1/8		0/8	
	0.011	1/8		0/8	
	0.004	0/8		0/8	
H33-58HI	0.300	8/8	0.053	8/8	0.049
	0.100	7/8		8/8	
	0.033	1/8		1/8	
	0.011	1/8		1/8	
	0.004	0/8		0/8	
H58-33BC	0.300	7/8	0.043	8/8	0.019
	0.100	6/8		8/8	
	0.033	4/8		7/8	
	0.011	0/8		1/8	
	0.004	1/8		0/8	
HPV58	0.300	0/8	>0.3	8/8	0.006
	0.100	0/8		8/8	
	0.033	0/8		7/8	
	0.011	0/8		6/8	
	0.004	0/8		3/8	

BALB/c mice(n=8 per dosage) were intraperitoneally inoculated once with the indicated doses (0.3, 0.1, 0.033, 0.011, 0.004μg) of H33-58HI VLP, H58-33BC VLP or HPV33 VLP, HPV58 VLP as control and bled 4 weeks later for antibody response. All VLPs were formulated with aluminum adjuvant.

* The ED_{50} values for HPV33 and HPV58 were calculated according to the method by Reed and Muench[5].

Supplementary Table 5. Half-Effective Dose (ED_{50}) in Mice for Aluminum Adjuvant-Containing Triple-Type Chimeric VLP or WT VLPs.

Antigen (VLP)	Dose (μg)	PsV33		PsV58		PsV52	
		Seroconversion no./ inoculated no.	ED_{50} (μg)*	Seroconversion no./ inoculated no.	ED_{50} (μg)	Seroconversion no./ inoculated no.	ED_{50} (μg)
H33-58HI-52DE	0.900	7/8	0.083	8/8	0.069	8/8	0.557
	0.300	7/8		8/8		4/8	
	0.100	6/8		6/8		2/8	
	0.033	0/8		0/8		0/8	
	0.011	0/8		0/8		0/8	
	0.004	0/8		0/8		0/8	
H33-58HI-52FG	0.900	8/8	0.046	5/8	0.243	7/8	0.087
	0.300	8/8		5/8		6/8	
	0.100	7/8		2/8		4/8	
	0.033	3/8		1/8		4/8	
	0.011	0/8		0/8		0/8	
	0.004	0/8		0/8		0/8	
H58-33BC-52DE	0.900	8/8	0.009	8/8	0.014	7/8	0.341
	0.300	8/8		7/8		4/8	
	0.100	7/8		7/8		0/8	
	0.033	5/8		4/8		0/8	
	0.011	6/8		6/8		0/8	
	0.004	2/8		1/8		0/8	
H58-33BC-52HI	0.900	8/8	0.065	8/8	0.041	8/8	0.058
	0.300	8/8		8/8		8/8	
	0.100	5/8		7/8		6/8	
	0.033	2/8		3/8		2/8	
	0.011	0/8		1/8		0/8	
	0.004	0/8		0/8		0/8	
HPV33	0.300	8/8	0.013	0/8	>0.3	0/8	>0.3
	0.100	7/8		0/8		0/8	
	0.033	6/8		0/8		0/8	
	0.011	5/8		0/8		0/8	
	0.004	0/8		0/8		0/8	
HPV58	0.300	1/8	>0.3	8/8	0.021	0/8	>0.3
	0.100	0/8		7/8		0/8	
	0.033	0/8		7/8		0/8	
	0.011	0/8		1/8		0/8	
	0.004	0/8		0/8		0/8	

HPV52	0.300	0/8	>0.3	0/8	>0.3	8/8	0.046
	0.100	0/8		0/8		7/8	
	0.033	0/8		0/8		3/8	
	0.011	0/8		0/8		0/8	
	0.004	0/8		0/8		0/8	
HPV33/52/58	0.300/0.300/0.300	6/8	0.009	6/8	0.009	6/8	0.017
	0.100/0.100/0.100	8/8		8/8		8/8	
	0.033/0.033/0.033	6/8		5/8		5/8	
	0.011/0.011/0.011	7/8		8/8		5/8	
	0.004/0.004/0.004	1/8		0/8		0/8	

Supplementary Table 6. Summarized Neutralization Titers of Immune Sera (at Week 8) in BALB/c Mice for Groups 1, -2, -3, -5 and -6 Immunized with 5 μg/Dose of Chimeric and WT L1 Proteins in Aluminum Adjuvant at Weeks 0, 2 and 4.

Immunogen (VLPs)		Neutralization Titer (log)		
		anti-typeA	anti-typeB	anti-typeC
Group 1	HPV52 (typeA)	6.0	0.2	0.2
	HPV33 (typeB)	0.2	4.8	1.2
	HPV58 (typeC)	0.2	2.0	5.5
	HPV58-33BC-52HI	4.3	4.6	4.8
	HPV52&33&58	5.4	4.6	5.2
Group 2	HPV16 (typeA)	5.3	2.0	0.4
	HPV31 (typeB)	1.8	4.8	0.4
	HPV35 (typeC)	0.4	0.4	5.4
	HPV35-16FG-31BC	4.8	4.3	4.4
	HPV16&31&35	4.6	4.7	5.3
Group 3	HPV66 (typeA)	4.4	1.7	0.8
	HPV56 (typeB)	1.3	5.1	0.4
	HPV53 (typeC)	0.4	0.4	4.3
	HPV66-56HI-53FG	4.1	3.9	3.6
	HPV66&56&53	4.5	5.0	3.8
Group 5	HPV68 (typeA)	4.7	0.4	1.2
	HPV39 (typeB)	0.4	4.6	0.4
	HPV70 (typeC)	1.6	0.7	4.3
	HPV39-68FG-70HI	4.3	3.9	3.5
	HPV68&39&70	4.5	4.8	4.2
Group 6	HPV59 (typeA)	5.1	1.6	0.4
	HPV45 (typeB)	0.4	5.2	1.6
	HPV18 (typeC)	1.6	0.4	5.3
	H45-18BC-59HI	4.0	4.3	4.4
	HPV59&45&18	5.2	5.1	5.1

Note: immune sera were drawn at week 8 in BALB/c mice immunized with 5 μg/dose of chimeric and WT L1 proteins in aluminum adjuvant at weeks 0, 2 and 4.

Supplementary Table 7. Binding kinetics of mAb 4E5 against HPV33 pent/HPV58 pent/ HPV52 pent and HPV chimeric pentamers.

Construct (pentamer)	Binding kinetics of pentamer:4E5 complex			
	$k_a(10^5M^{-1}\cdot s^{-1})$	$k_d(10^{-3}s^{-1})$	$K_A(10^7M^{-1})$	$K_D(10^{-8}M)$
HPV33	1.17±0.03	4.42±0.02	2.65±0.06	3.78±0.08
HPV58	NA	NA	NA	NA
HPV52	NA	NA	NA	NA
H58-33BC	0.68±0.05	4.16±0.05	1.62±0.10	6.16±0.38
H58-33BC-52HI	1.02±0.07	5.11±0.11	1.99±0.11	5.02±0.27

Supplementary Table 8. Cryo-EM Data Collection and Reconstruction.

VLP:Fab complex	HPV33:4E5	HPV58-33BC:4E5	HPV58-33BC-52HI:4E5
Data Collection			
EM equipment		Tecnai F30	
Voltage		300 KV	
Detector		Falcon II	
Pixel size (Å/pixel)		1.128	
Electron dose (e-/Å^2)		25	
Defocus range (μm)	0.48-3.67	1.18-3.65	0.50-4.48
Reconstruction			
Software		AUTO3DEM-4.05.2<br>EMAN2<br>RELION2.0	
Number of particles picked from micrographs	5,117	2,519	6,126
Number of particles used for reconstruction	753	723	908
Final resolution (Å)	12.3	12.0	10.0

Supplementary References

1. Li Z*, et al.* Crystal Structures of Two Immune Complexes Identify Determinants for Viral Infectivity and Type-Specific Neutralization of Human Papillomavirus. *mBio* **8**, (2017).
2. Bishop B*, et al.* Crystal structures of four types of human papillomavirus L1 capsid proteins: understanding the specificity of neutralizing monoclonal antibodies. *The Journal of biological chemistry* **282**, 31803-31811 (2007).
3. Li Z*, et al.* The C-Terminal Arm of the Human Papillomavirus Major Capsid Protein Is Immunogenic and Involved in Virus-Host Interaction. *Structure* **24**, 874-885 (2016).
4. Xiao C, Rossmann MG. Interpretation of electron density with stereographic roadmap projections. *Journal of structural biology* **158**, 182-187 (2007).
5. Reed LJ MH. A simple method of estimating fifty percent endpoints. *Am J Hyg* **27**, 493-497 (1938).
